# An artificial neural network approach for predicting hypertension using NHANES data

**DOI:** 10.1038/s41598-020-67640-z

**Published:** 2020-06-30

**Authors:** Fernando López-Martínez, Edward Rolando Núñez-Valdez, Rubén González Crespo, Vicente García-Díaz

**Affiliations:** 1 Department of Computer Science, Oviedo University, C/ Federico Garca Lorca, 33007 Oviedo, Spain; 2 Department of Computer Science and Technology, Universidad Internacional de La Rioja, Av. de la Paz, 137, 26006 Logroño, La Rioja Spain; 3 Sanitas, 8400 NW 33rd St, Doral, FL 33122 USA

**Keywords:** Computer science, Information technology, Medical research

## Abstract

This paper focus on a neural network classification model to estimate the association among gender, race, BMI, age, smoking, kidney disease and diabetes in hypertensive patients. It also shows that artificial neural network techniques applied to large clinical data sets may provide a meaningful data-driven approach to categorize patients for population health management, and support in the control and detection of hypertensive patients, which is part of the critical factors for diseases of the heart. Data was obtained from the National Health and Nutrition Examination Survey from 2007 to 2016. This paper utilized an imbalanced data set of 24,434 with (69.71%) non-hypertensive patients, and (30.29%) hypertensive patients. The results indicate a sensitivity of 40%, a specificity of 87%, precision of 57.8% and a measured AUC of 0.77 (95% CI [75.01–79.01]). This paper showed results that are to some degree more effectively than a previous study performed by the authors using a statistical model with similar input features that presents a calculated AUC of 0.73. This classification model can be used as an inference agent to assist the professionals in diseases of the heart field, and can be implemented in applications to assist population health management programs in identifying patients with high risk of developing hypertension.

## Introduction

Currently, the use of neural network models for disease classification is increasing rapidly, not only because of a significant amount of data available that is being generated by healthcare devices and systems but also for the magnitude of computational resources available for data calculation and processing^1, 2^. This immense volume of data is utilized to train models importantly, and facilitates the use of expert systems, NLP techniques^3, 4^ and classification techniques for finding trends and patterns in the evaluation and classification of several diseases. Hypertension is considered in the group of risk factors for cardiovascular disease that caused 17.7 million deaths in the world in 2015^5–7^. In the United States, hypertension is contemplated as the primary determinant of decease among U.S. adults even with the existence of practical and low-cost treatments^8–10^, with significant public health risks and economic implications for U.S. population. The National Health and Nutrition Examination Survey (NHANES) conducted by the National Center for Health Statistics is one of the principal sources for tracking hypertension in the U.S. population^11^ with vast amounts of features and data related to cardiovascular diseases.

In this paper, we develop a neural network classification model to predict hypertension with non-invasive risk factors applying healthcare data from the NHANES. A multi-layer neural network architecture was used to identify hypertensive patients at risk of developing hypertension. Our primary goal in this paper was to train a classifier that will identify hypertensive patients in a highly imbalanced NHANES data set. Additionally, we aspire to achieve lower error rate with a neural network architecture compared to the logistic regression model developed in a previous paper^12^ by using the same set of input features. The motivation to develop a new model was the non-linearity of the input features, and neural networks are usually trained to treat non-linearity due to the non-linear nature of them^13^, making the model more flexible compared to logistic regression.

This paper is organized along these sections. Second section describes related work and literature research of various models that implemented neural networks for cardiovascular disease classification. Third section introduces the elaboration of the model, population, data source, and validation. Fourht section combines statistical and clinical analysis. Fifth section presents our results and limitations. Finally, sixth section presents conclusions and future work.

## Related work

We reviewed a few published papers adopting neural networks models to infer hypertension, and some other studies that compared classification performance and accuracy with logistic regression^14–16^. In every paper, the development process, feature selection, ground truth definition, training data sets, test data sets, overfitting prevention, error assessment, and accuracy information were reviewed. We also reviewed if the models were validated or not, either by an unseen data set or by a panel of experts in the domain^17–20^.r Some of the models are shown in Table 1.

**Table 1:** Related work.

Author	Input features	n Total	Type of model	AUC (%)
**Artifical neural network models comparison**				
LaFreniere et al.^21^	Age, gender, BMI, sys/diast BP, high and low density lipoproteins, triglycerides, cholesterol, microalbumin, and urine albumin creatinine ratio	379,027	Backpropagation neural network	82
Polak and Mendyk^22^	Age, sex, diet, smoking and drinking habits, physical activity level and BMI	159,989	backpropagation (BP) and fuzzy network	75
Tang et al.^23^	Sys/diast BP, fasting plasma glucose, age, BMI, heart rate, gender, WC, diabetes, renal profile	2,092	Feed-forward, back-propagation neural network	76
Ture et al.^24^	Age, sex, hypertension, smoking, lipoprotein, triglyceride, uric acid, total cholesterol, BMI	694	Feed-forwardneural network	81
Lynn et al.^25^	Sixteen genes, age, BMI, fasting blood sugar, hypertension medication, no history of cancer, kidney, liver or lung	22,184 genes, 159 cases	One-hidden-layer neural network	96.72
Sakr et al.^6^	Age, gender, race, reason for test, stress, medical history	23,095	Backpropagation neural network	64
López-Martínez et al.^12^	Age, gender, ethnicity, BMI, smoking history, kidney disease, diabetes	24,434	Three-hidden layer neural network	77

LaFreniere et al.^21^ presented an artificial neural network (ANN) to predict hypertensive patients utilizing the Canadian Primary Care Sentinel Surveillance Network (CPCSSN) data set. The independent features used were age, gender, BMI, systolic and diastolic blood pressure, high and low-density lipoproteins, triglycerides, cholesterol, microalbumin, and urine albumin–creatinine ratio. Confusion matrix and Receiver Operating Characteristic (ROC) curve were utilized to measure the accurateness of the model. This paper used an extensive data set to train the model compared with other studies.

Polak and Mendyk^22^ improve and validate an artificial neural network for high blood pressure risk, using data from the Center for Disease Control and the National Center for Health Statistics (CDC-NCHS). The independent features used in this model were age, sex, diet, smoking and drinking habits, physical activity level and BMI index. ROC curve was utilized to measure the accurateness of the model, and they performed a comparison with a logistic regression classification model.

Tang et al.^23^ presented an artificial neural network for classification of cardiovascular disease including hypertension; this paper used a Chinese population. Statistical analysis indicated that 14 risk factors showed statistical importance with cardiovascular disease. The ROC curve is utilized to measure the performance of the model.

Ture et al.^24^ implemented a multilayer perceptron for the classification of hypertensive patients. The independent features utilized were age, sex, family history of hypertension, smoking habits, lipoprotein, triglyceride, uric acid, total cholesterol, and body mass index (BMI). ROC curve is utilized to measure the accuracy of the model.

Lynn et al.^25^ constructed a neural network model to simulate the geneendophenotype-disease relationship for Taiwanese hypertensive males. Sixteen genes, age, BMI, fasting blood sugar, hypertension medication, no history of cancer, kidney, liver or lung. Classification accuracy is utilized to measure the performance of the model.

Sakr et al.^6^ built an artificial neural network to compares the performance of it with different machine learning techniques on predicting the risk of developing hypertension. Age, gender, race, a reason for the test, stress tests and medical history used for classification. ROC curve is utilized to measure the accuracy of the model.

We identified other studies for predicting hypertension using ANN, and all of them have advantages and disadvantages. However, the above mentioned are the most relevant. Our paper did not use data from any medical facility as the studies mentioned earlier. However, our model used a data sample more significant that the majority of them, collected from a national examination survey. The number of predictors was small and non-invasive, in comparison with the cited studies that used lab and exam data.

The national examination survey was designed to assess the health and nutritional status of adults and children in the United States, the data on this survey is unique because it combines social determinants of health data such as smoking, alcohol consumption and dietary habits, and physical examinations. This survey emphasized data regarding the prevalence of major diseases and risk factors for diseases for a broader population than just data from a medical facility, which contains only data for a small subset of the population that does not represent the entire picture of significant disease. In addition, historically, disease trends in the United States have been assessed by surveys.

We achieve an AUC of 0.77 which is acceptable and close to all the studies, considering the imbalanced data used in our paper. The results in our paper and the cited studies could be successfully utilized in hypertension classification, and can be included as inference engines in expert systems for hypertension screening tools. Our paper also includes more hidden layers that the others and we determined the number of hidden layers through cross-validation experiments. Not evidence of the number of layers and hidden nodes selection techniques are present in the studies.

## Materials and methods

We present and discuss an alternative approach, using artificial neural network to classify hypertensive patients. We build, trained, and evaluated the model with the Sklearn of Python programing language package^26^, Microsoft Cognitive Toolkit (CNTK) from Microsoft, and Azure Machine Learning Studio^27, 28^.

A cross-sectional analysis comes from the collection of health examination data for a representative sample of noninstitutionalized U.S. residents, questionnaires administered in the home of the residents. The interview collects demographic, health, and nutrition information, as well as information about the household. This examination includes physical measurements such as blood pressure, dental examination, and the collection of blood and urine specimens for lab testing.

### Data source

We collected NHANES data sets from NHANES 2007–2008 to NHANES 2015–2016. This dataset is intended for public access and health care utilization. This datasets are prepared and published through the Centers for Disease Control and Prevention (CDC) to provide full access. Statistic characterizing human populations, laboratory data, blood pressure, body measures data and questionnaires linked to diabetes, smoking, and kidney conditions are part of the data set. The original data set consists of five folders from 2007 to 2016, each one of them contains a pdf file with statistics of the response rates of the NHANES survey and the SAS Transport files for all the survey measure variables. After imported the original data sets in python, data extraction and transformation were necessary to select and categorize the input features. We created a repository in Github with the original files from NHANES, the final data set used to run the model and the notebooks used for the data preparation^29^.

### Ethic review board approval

For the use of NHANES data, the Institutional Review Board (IRB) approval and documented consent was obtained from participants. The description of the survey name and data, and the NCHS IRB/ERB Protocol Number or Description can be found in Centers for Disease Control and Prevention^30^. In 2003, the NHANES Institutional Review Board (IRB) changed its name to the NCHS Research Ethics Review Board (ERB). The National Center for Health Statistics (NCHS) offered downloadable public-use data files through the Centers for Disease Control and Preventions (CDC) FTP file server. NHANES survey is a public-use data files prepared and disseminated to provide access to the full scope of the data. This allows researchers to manipulate the data in a format appropriate for their analyses. In our study, by using these data we signify our agreement to comply with the data use restrictions to ensure that the information will be used solely for statistical analysis or reporting purposes. The data use restrictions can be found in National Center for Health Statistics^31^. In this study, all experiments were performed in accordance with relevant guidelines and regulations.

### Study population and analysis

Healthcare survey data collected in the course of 2007–2016 was used to train and evaluate the classification model. A neural network model was developed to assess the importance of several factors and their relation with prevalence of hypertension with a symbolical sampling of adults $\ge$ 20 years in the United States (n = 24,434). Table 2 shows the grouping of the hypertensive patients by race and gender.

**Table 2:** n samples by hypertensive class, gender and race.

Class	Gender	Race	n
**Hypertension, adults 20 and over: 2007–2016**			
Hypertensive	Female	Mexican American	464
		Non-Hispanic black	925
		Non-Hispanic white	1,433
		Other Hispanic	368
		Other race—including multi-racial	277
	Male	Mexican American	575
		Non-Hispanic black	1,039
		Non-Hispanic white	1,582
		Other Hispanic	371
		Other race—including multi-racial	365
Non-hypertensive	Female	Mexican American	1,461
		Non-Hispanic black	1,676
		Non-Hispanic white	3,663
		Other Hispanic	1,084
		Other race—including multi-racial	1,038
	Male	Mexican American	1,275
		Non-Hispanic black	1,465
		Non-Hispanic white	3,585
		Other Hispanic	820
		Other race—including multi-racial	968
		Total	24,434

### Input features

Several studies in the US integrated healthcare system in cardiovascular research with incident hypertension showed association between race, age, smoking, BMI, diabetes, and kidney conditions with hypertension^32–34^. Among different cohorts of patients with hypertension, during the follow up, individuals present more kidney disease, diabetes problems and remarkable association with smoking habits. In addition, these studies shown that effective BMI management decrease the incidence of hypertension, hypertension prevalence increase with age, and race is a significant factor of prevalence of hypertension.

For this paper, and based on the previous analysis, the selected input features are race, age, smoking, body mass index (BMI), diabetes, and kidney conditions. Participants admit to have diabetes if the answer presents “Yes or “Borderline” to the question “Doctor told that you have diabetes?”^35^. Smokers defined as individuals having smoked $\ge$ 100 cigarettes during their lifetime, and currently smoke some days or every day^36^. Chronic kidney disease (CKD) defined as “yes” response to the question “Have you ever told by a health care provider you have weak or failing kidneys?” during the interview, and for NHANES 2015–2016, CKD defined as a glomerular filtration rate (GFR) $\le$ 60 ml/min/1.73 $\hbox {m}^{2}$^37^ and albumin–creatinine ratio $\ge$ 30 mg/g^38^. Body mass index and age transformed from continues features to Categorical features to understand the relationship among the features. Blood pressure is utilized to generate the dependent feature.Hypertension category designated as systolic blood pressure of $\ge$ 130 mmHg, previously define as $\ge$ 140 mmHg by the American Heart Association^39^. Table 3 show the independent features and the dependent feature.

**Table 3:** Variables included in the model.

Variable name	Description	Code	Meaning
Gender	Gender	1	Male
		2	Female
Agerange	Age at screening adjudicated—date of birth was used to calculate AGE	1	20-30
		2	31–40
		3	41–50
		4	51–60
		5	61–70
		6	71–80
Race	Race/Hispanic origin	1	Mexican American
		2	Other Hispanic
		3	Non-Hispanic white
		4	Non-Hispanic black
		5	Other race—including multi-racial
BMXBMI	Body mass index (kg/m$^{2}$)	1	Underweight = $<18.5$
		2	Normal weight = 18.5–24.9
		3	Overweight = 25–29.9
		4	Obesity = BMI of 30 or greater
Kidney	Ever told you had weak/failing kidneys	1	Yes
		2	No
Smoke	Smoked at least 100 cigarettes in life	1	Yes
		2	No
Diabetes	Doctor told you have diabetes	1	Yes
		2	No
		3	Borderline
Hypclass	Systolic: blood pres (mean) mm Hg	0	Non-hypertensive
		1	Hypertensive

### Features selection

Clinical importance was pertinent plus the statistical significance of the features to choose the final inputs. For this paper, we utilized chi-square because previous work indicates that this statistical test performs well to evaluate sets of categorical features^40–43^. Some heuristic methods were investigated to confirm the usefulness and relevance of the features. Genetic algorithm with other machine learning methods probably generates better results^42^, and produce adequate time complexity to find optimal solutions^44, 45^. We will consider it and discuss it in forthcoming studies. For this paper, we utilized statistical methods between each feature due to the nature of the inputs. Table 4 shows all features with their *p* values and scores. Based on the clinical significance of all input features, our clinical expert decided not to exclude any variable from the paper due to the relationship among features in previous studies. In addition, one of the options we used to get the feature importance or the influence of a given parameter in the classification model is to obtain and examine the coefficient of the parameters with the provided dataset as shown in López-Martínez et al.^12^ Coefficients and Odds Ratio. Features using the same scale with larger coefficients are more important because they represent more significant changes in the dependent variable.

**Table 4:** Chi-squared between each variable.

Feature	*p* value	Score
Gender	0.3988107	0.711909
Agerange	0.000000	1,965.607023
Race	0.008822	6.858521
BMIrange	0.0172385	5.67193
Kidney	0.3546428	0.856775
Smoke	0.0975246	2.745566
Diabetes	0.0012164	10.465222

### Neural network model

An artificial neural network describes a machine learning algorithms that are made of layers of nodes, usually, an input layer, hidden layers^46^ and an output layer^47^. Figure 4 shows the form of the neural network architecture developed for this paper. The input nodes values are encoded and normalized with gaussian normalization^48^ in order to improve the computation.

### The non-linearity of the model

**Fig. 1:**
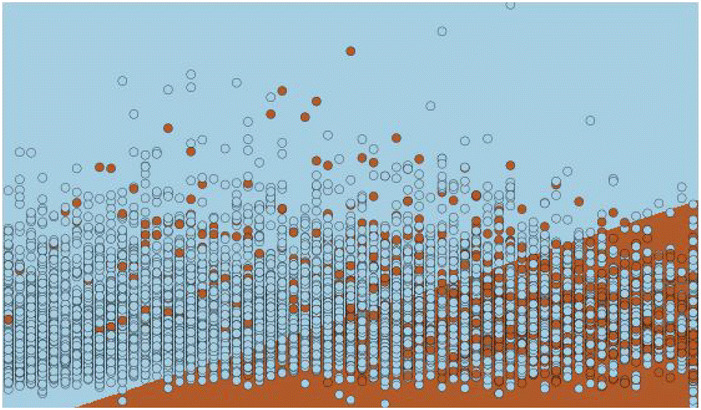
Decision boundary.

**Fig. 2:**
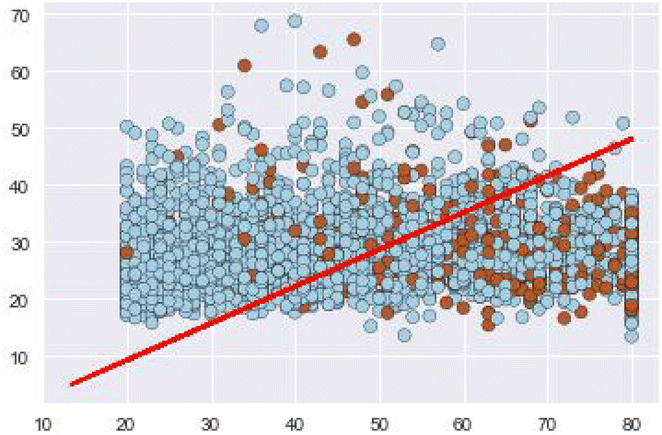
Draw test points.

The motivation of developing this neural networks model is the ability to use non-linear activation functions to eliminate the non-linearity of the input features. The data used to train the model is not linearly separable, this means that there is no line that separates the data points easily as shown in Figs. 1 and 2 where we plot several input variables and the decision boundary using logistic regression.

**Fig. 3:**
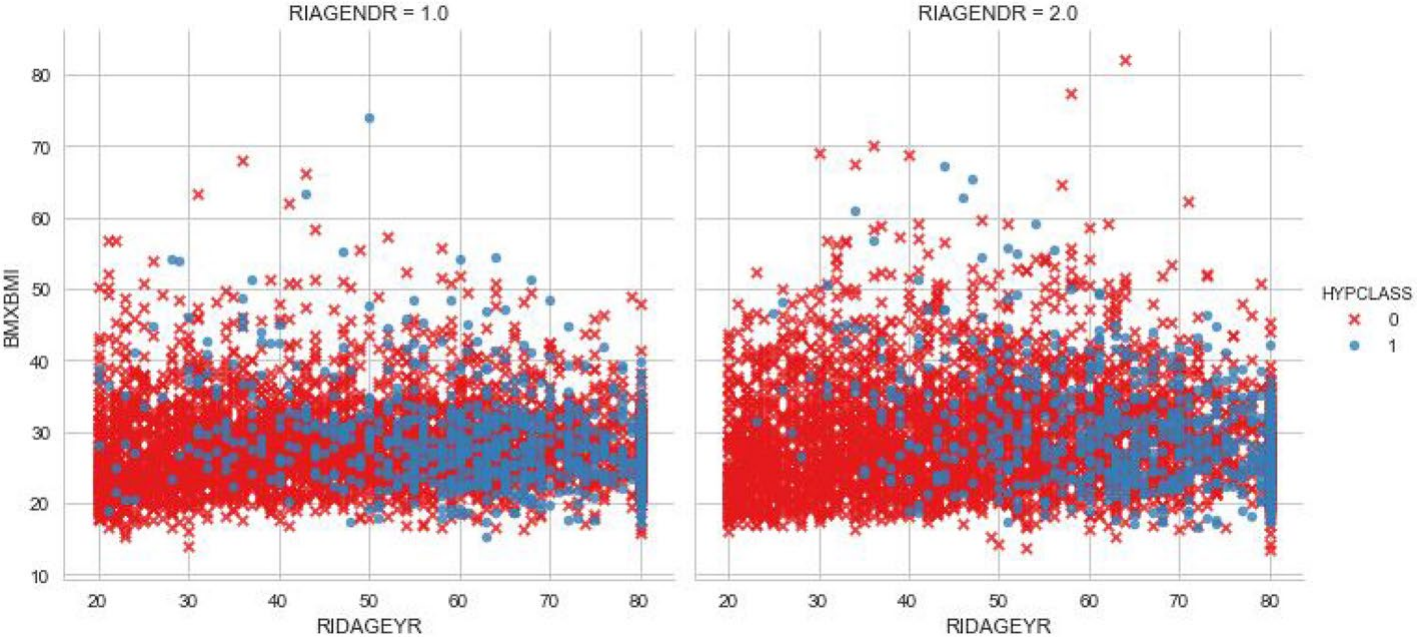
Relation between BMI and age by gender and hypertension class.

This non separability can be seen also if we plot three input variables such as gender, age and bmi as shown in Fig. 3. The Neural network model learn a new representations of the data which makes the classification more approachable with respect to this representation. A non-linear activation function allows non-linear classification with a non-linear decision boundary which will be a hyperplane that is orthogonal to the line.

**Fig. 4:**
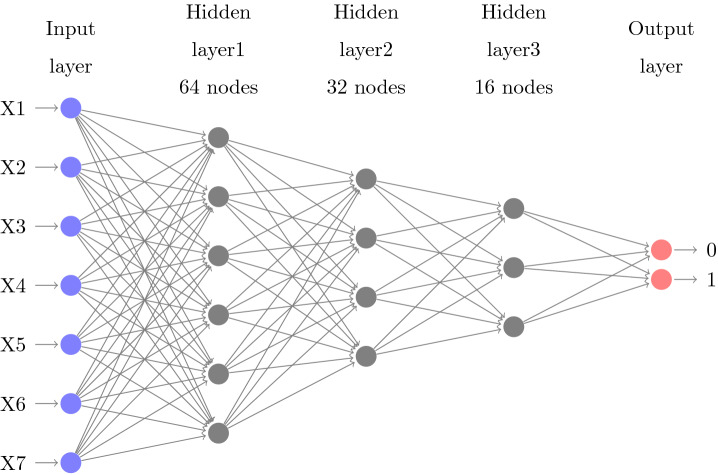
Multilayer perceptron architecture.

### Model training

The generated probabilities need to be as proximate as possible to the observed features. The loss function calculated as the difference between the learned model against the generated by the training set^49^. Cross-entropy with Softmax is utilized, but the mathematical computation of the derivative is not presented in this paper^50^ (Fig. 4).

**Fig. 5:**
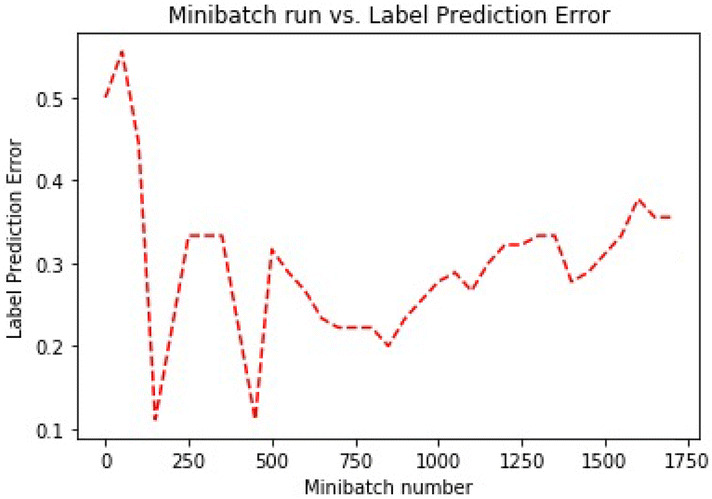
Training error.

**Fig. 6:**
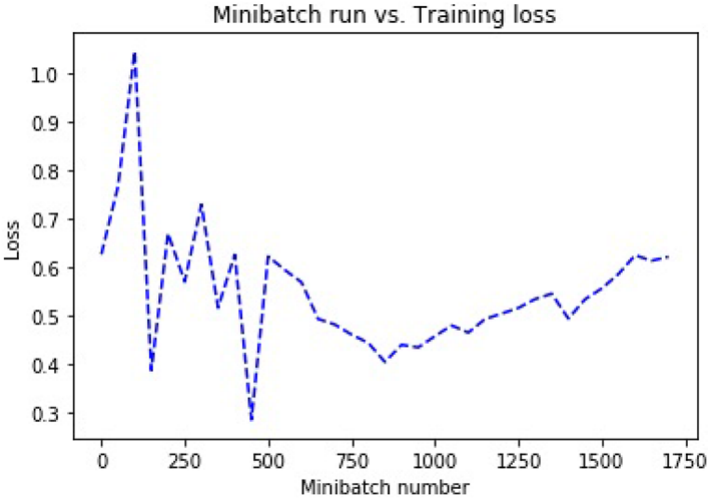
Loss error.

Random initialization of the parameters is the first step, and the network produces a new set of parameters after each evaluation. In this network, He initialization^51^ is utilized. This type of initialization is comparable with the Xavier initialization excepting Xavier uses a different weights scaling factors *W* in layer *l*, and the author recommended for layers with ReLU activation. Mini-batches are utilized to train the model. Learning rate^52^ is a factor that balances how much the parameters change in every iteration. Each iteration works on ten samples, and the model is trained on 70% (17,104) of the data set. Table 5 presents the parameters of the architecture, and Figs. 5 and 6 present the training loss and classification error of the mini-batch run for the model.

**Table 5:** Model architecture parameters.

Parameter	Value	
Input dimension	7	
Num output classes	2	
Num hidden layers	3	
Hidden layer1 dimension	64	
Activation func layer1	Relu	
Hidden layer2 dimension	32	
Activation func layer2	Relu	
Hidden layer3 dimension	16	
Activation func layer3	Relu	
Minibatch size	10	
Num samples to train	17,104	
Num minibatches to train	1,710	
Loss function	Cross entropy with softmax	
Eval error	Classification error	
Learner for parameters	Momentum sgd	
Learning rate	0.01	
Momentum	0.9	
Eval metrics	Confusion matrix, AUC	

### Model evaluation

To evaluate the classification model, computation of the average test error is utilized. The algorithm finds the position of the highest value in the output array, and compares it to the actual label. The evaluation of the network is performed on data never used for training, and coincide with the 30% (7,331) of the data set. The resulting error is compared with the training error and the results indicates that the model presents a useful generalization error. Our model can meritoriously deal with unseen observations, and this is one of the keys to avoiding overfitting^53^.

**Fig. 7:**
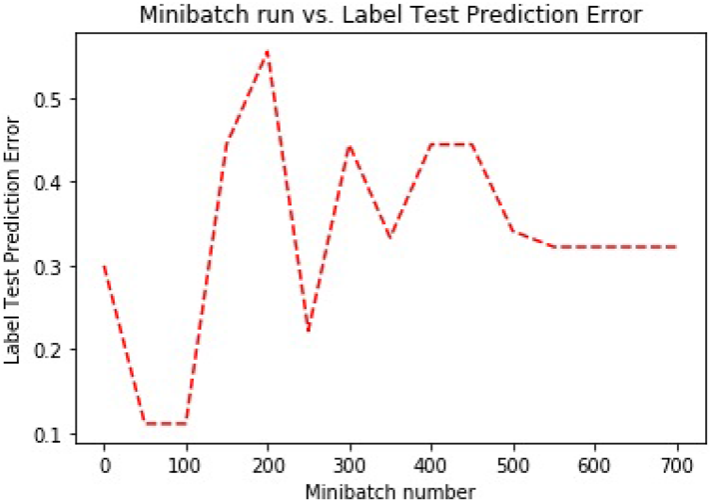
Test prediction error.

For each observation, our model use softmax as the evaluation function that returns the probability distribution across all the classes. In our paper, it would be a vector of 2 elements per observation. The output nodes in our model convert the activations into a probability and map the aggregated activations across the network to probabilities across the two classes. Figure 7 shows the test classification error for our model.

In this paper, we utilized a few evaluation metrics to evaluate the model. Results are show in Table 6. Summary of the actual label versus the predicted. True positive value (887), True Negative value (4,477), False Negative (1,318) and False Positive value (648).

**Table 6:** Confusion matrix.

	Predicted	
	Non-hypertensive	Hypertensive
**True**		
Non-hypertensive	4,477	648
Hypertensive	1,318	887

Table 7, shows the classification report with the sensitivity, precision, and the harmonic mean. The low precision and sensitivity on the hypertensive label is caused because of the large presence of false positives, and imbalanced of the testing data set.

The sensitivity of the model moderately acceptable due to the imbalanced testing data set, and this reveals a high number of false negatives.

**Table 7:** Classification report.

True positive	False negative	Precision	Accuracy
887	1,318	0.578	0.732
False positive	True negative	Recall	f1-score
648	4,477	0.402	0.474
Positive label: 1		Negative label: 0	

## Comparison of the model with alternative techniques

Conducting data analysis in a highly imbalanced data set is not trivial and often leads to obtain low sensitivity results. A comparison of our proposed ANN with other highly interpretable methods will allow us to compare the AUC curves of the models and to validate the performance and sensitivity of disease diagnostic. Five machine learning algorithms were identified and compared using the NHANES data. These algorithms not only accurately classify hypertensive patients, but were able to identify key features useful for hypertension diagnostic. A Two-Class Decision Jungle that represents an ensemble of decision directed acyclic graphs (DAGs), a Two-Class Boosted Decision Tree, an ensemble learning method. A Two-Class Bayes Point Machine, that efficiently approximates the Bayesian average of linear classifiers by choosing one “average” classifier, the Bayes Point. A Two-Class Support Vector Machine, and a Two-Class Logistic Regression.

### Two-class decision jungle

This algorithm is a development of decision forest that lie on ensemble of decision directed acyclic graphs (DAGs), used to obtain accurate classifiers^54^. Decision jungles are very comparable to random forests, but it uses DAGs instead of trees as base learners. This structure is more memory-efficient because it eliminates the need for repeating leaf nodes, but it needs more computing time.

This method is selected because decision jungles are models that can express non-linear selection boundaries, and they are strong in the existence of noisy features. Table 8 shows the parameters and Table 14 shows the classification report.

**Table 8:** Decision jungle parameters.

Parameter	Value	
**Two-class decision jungle parameters**		
Resampling method	Bagging	
Trainer mode	Single parameter	
Number of decision DAGs	8	
Maximum depth of the decision DAGs	32	
Maximum width of the decision DAGs	128	
Number of optimization steps per layer	2,048	

### Two-class logistic regression

Logistic regression is a well-known classification technique especially used for classification tasks. The algorithm tries to find the optimal values by maximizing the log probability of the parameters given the inputs. Maximization is performed by using a method for parameter estimation called Limited Memory BFGS^55^. Table 9 shows the parameters and Table 14 shows the classification report.

**Table 9:** Logistic regression parameters.

Parameter	Value	
**Two-class logistic regression parameters**		
Optimization tolerance	1.00E−07	
L1 regularization weight	1	
L2 regularization weigh	1	
Memory size for L-BFGS	20	

### Two-class support vector machine

The Support Vector Machine algorithm is a supervised learning model that evaluates input data in a multi-dimensional label zone called the hyperplane. The inputs are points in this zone or space, and are mapped to outputs that are divided as clear as possible^56^. Table 10 shows the parameters and Table 14 shows the classification report.

**Table 10:** Support vector machine parameters.

Parameter	Value	
**Two-class support vector machine parameters**		
Lambda—weight for L1 regularization	1.00E−03	
normalize features before training	Yes	

### Two-class boosted decision tree

A boosted decision tree, ensemble learning method where the trees corrects for the errors of the previous trees. Predictions are established on the full ensemble of trees^57^. Table 11 shows the parameters and Table 14 shows the classification report.

**Table 11:** Boosted decision tree parameters.

Parameter	Value	
**Two-class boosted decision tree parameters**		
Maximum number of leaves per tree	20	
Minimum number of training instances	10	
Learning rate	0.2	
Number of trees constructed	100	

### Two-class Bayes point machine

This method approximates the optimal Bayesian average of linear classifiers by choosing the Bayes Point. This method created by Microsoft Research has shown that no external hyper-parameters are needed and the model can be trained in a single pass, without over-fitting, and without needing pre-processing steps such as data re-scaling^58^. Table 12 shows the parameters and Table 14 shows the classification report.

**Table 12:** Bayes point machine parameters.

Parameter	Value	
Number of training iterations	30	
bias to be added to each instance in training	Yes	

### Synthetic minority oversampling technique

In addition to the previous methods, we have decided to solve the imbalance problem by using the Synthetic Minority Oversampling Technique (SMOTE) and compare the performances with all the methods. This statistical technique increase the number of underrepresented cases in the dataset used in the study. This method returns a dataset that contains the original samples, plus an additional number of synthetic minority samples. In our case we have increase the number of cases 200% (module doubles the percentage of minority cases compared to the original dataset) and the number of nearest neighbors used was 5 (A nearest neighbor is a a case that is very similar to some target case. The distance between any two cases is measured by combining the weighted vectors of all features)^59^. Increasing the number of cases using this technique is not guaranteed to produce more accurate results. We experimented with different percentages, different feature sets, and different numbers of nearest neighbors to find the best results. Table 13 shows the parameters and Table 14 shows the classification report.

**Table 13:** Synthetic minority oversampling parameters.

Parameter	Value	
SMOTE percentage	200	
Number of nearest neighbors	5	

Table 15 presents the result of comparing six methods with our ANN proposed method. We presented the AUC and the corresponding accuracy rates. We observed that the accuracy of the methods are very similar with imbalanced dataset, but the AUC and f1-score of our method are slightly higher and competitive; except for the technique used to solve the imbalance of the dataset which shows a higher AUC. however, no one is statistically better than the others. We utilized cross-validation to measure the performance of the models, and performed several train-score-evaluate operations (10 folds) on different subsets of the input data. An statistical significance test, proposed by Giacomini and White^60^ was utilized. Where the predictive ability of the presented model for the Cross-entropy loss function showed better performance than the others. We performed a pairwise test of predictive ability of the five models using the Cross-entropy loss function. Table 16 shows the results for the cases. The minus sign indicates that the model under performs the method in the row at the 5% significance level, evidenced by the value in parenthesis (critical value) greater than 1.

**Table 14:** Classification report.

Method	True positive	False negative	False positive	True negative	Precision	Accuracy	Recall	f1-score
Our model	887	1,318	648	4,477	0.578	0.732	0.402	0.474
Decision jungle	540	912	390	3,045	0.581	0.734	0.372	0.453
Logistic regression	557	895	389	3,046	0.589	0.737	0.384	0.465
Support vector machine	556	896	387	3,048	0.59	0.737	0.383	0.464
boosted decision tree	568	884	439	2,996	0.564	0.729	0.391	0.462
Bayes point machine	543	909	388	3,047	0.583	0.735	0.374	0.456
Synthetic minority oversampling	3,645	789	1,326	2,086	0.73	0.73	0.82	0.77
Positive label: 1					Negative label: 0

**Table 15:** Classification methods comparison.

Method	Precision	Accuracy	f1-score	AUC
Our model	0.578	0.732	0.474	0.77
Decision jungle	0.581	0.734	0.453	0.769
Logistic regression	0.589	0.737	0.465	0.764
Support vector machine	0.59	0.737	0.464	0.759
Boosted decision tree	0.564	0.729	0.462	0.765
Bayes point machine	0.583	0.735	0.456	0.763
Synthetic minority oversampling	0.73	0.73	0.77	0.8

**Table 16:** Predictive ability tests.

	DJ	LR	SVM	BDT	BPM
ANN	0.001- (3.65)	0.035- (1.80)	0.001- (4.03)	0.036- (1.80)	0.011- (1.67)

## Results and analysis

The classification of hypertensive patients was carried out by using artificial neural network with back-propagation. Statistical and clinical analysis were performed to explain the results.

### Statistical analysis

In our paper, we used a non-linear model to determine the unidentified non-linearity of the input. The test data set of 7,330 includes 5,125 (69.9%) non-hypertensive samples and 2,205 (30.,1%) hypertensive samples. The model shows a sensitivity of 887/2,205 = 40% (positives properly classified), and a specificity of 4,477/5,125 = 87% (negatives properly classified).

**Fig. 8:**
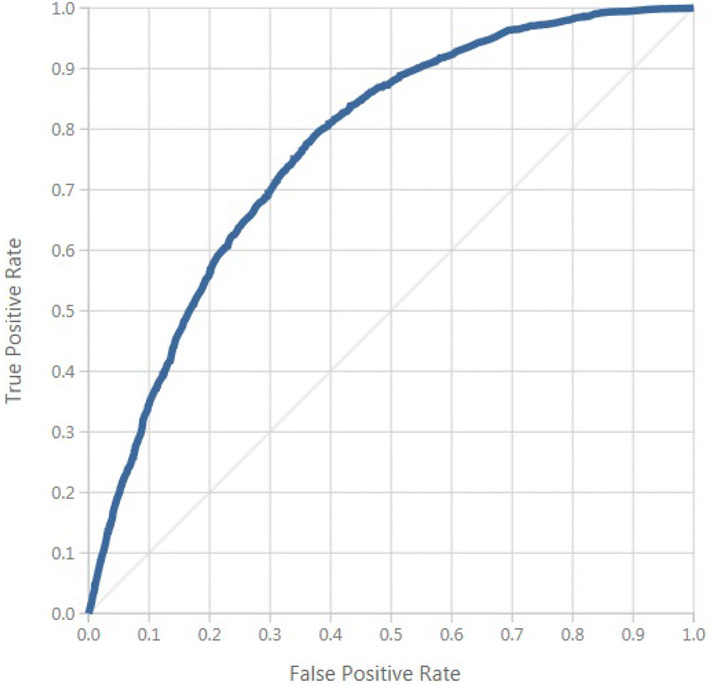
ROC curve.

**Fig. 9:**
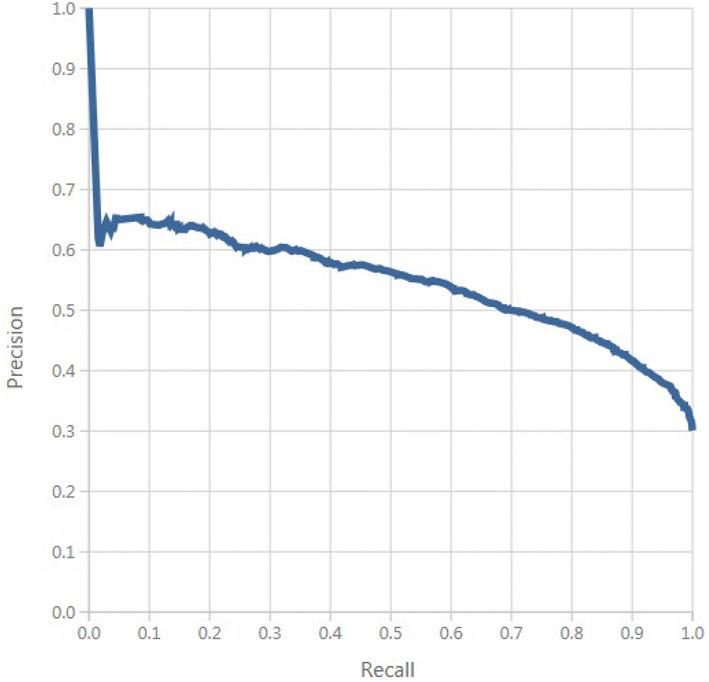
Precision/recall.

A positive predicted value of 887/1,535 = 57%, and a negative predicted value of 4,477/5,795 = 77%. A false negative rate of 1,318/2,205 = 59%, and a false positive rate of 648/5,125 = 12%. A false alarm of 12%, and a likelihood ratio for negative patients of 0.68%. In this paper, the multi-layer neural network model exceed at classifying patients who will not develop hypertension than those that will develop hypertension. The area under the curve is shown in Fig. 8. Figure 9 shows the proportion of the true results of overall positives results in contrast with the fraction of all correct results.

### Clinical analysis

With a sensitivity of 40%, and specificity of 87%, the artificial neural network model demonstrates that it might be ineffective for healthcare diagnosis in detecting positive occurrences, but the true negative rate demonstrates that the model is effective at finding non-hypertensive patients. The high negative predicted value of 77% shows that our model can be used as an examination tool. The positive cases of 57% shows that our model is superior to a random inference with a low probability for negative test results. This model correctly identifies non-hypertensive patients with an accuracy of 73%.

## Discussion and limitations

Even though a multi-layer neural network with one layer can model a vast variety of problems in the clinical domain, in our paper, a model with three hidden layers was advantageous to approximate the highly non-linear behavior of the input features. The result of the model was affected by the imbalanced data set, but we did not balance it to maintain the real distribution of the samples.

The current model configuration and size of the data source captured the complexity of the data. We used data augmentation to generate more input data from the already collected data, but the model was over-fitting and learned too many specific details about the training set. We reduced the number of training iterations to prevent over-fitting, and the accuracy was the same as the model without data augmentation.

The paper has shown that the classification capability of the model improved (AUC—0.77), based on the results of the statistical model utilized in a previous paper (AUC—0.73) when applied to the input features gender, race, BMI, age, smoking, kidney conditions and diabetes. However, challenges in applying artificial neural networks to the clinical domain remain. The use of deep learning to analyze hypertension risk features can be considered as complementary for the traditional approach and might be considered to validate other statistical models.

Our model achieved an AUC of 0.77 and used a smaller network architecture than the architecture used by Polak and Mendyk^22^ obtaining an AUC of 0.73 and^23^ that achieves an AUC of 0.76. However, our model presents a bigger network than^24^ that developed a network that achieves an AUC of 0.81, LaFreniere et al.^21^ achieves an AUC of 0.82 and Lynn et al.^25^ achieves an AUC of 0.96.

One of the significant limitations of our model is that it was developed using a highly imbalanced data set from the CDC to which a high prevalence of non-hypertensive patients was observed. There was no significant increase in accuracy. And we are not relying on this measure. We have a severe class imbalance, and the model will maximize accuracy by simply always picking the most common class.

Therefore, our model must be validated in other clinical settings, and further studies should include other neural network architectures and socio-demographic information^61^ to improve the precision and recall of the model, and to consider the integration of this model to the clinical diagnostic scheme. Also, adequate training data volume will be needed to train a bigger model to improve the classification results.

## Conclusions and future work

This paper presents a neural network approach to overthrown the non-linearity problem with the risk factors utilized as inputs for the model. This paper shows that the proposed model improves the accuracy and performance of a previous paper that used the same input features and the results were better than logistic regression in a small percentage. The main contribution of this paper is the developed model and based on results showed that ANN was the proper model compared with the previously developed LR model.

Our multi-layer model confirmed the influence of the imbalanced data set to the class with more presence in the data. This paper showed that the proposed model could be a guide to the design of other models and inference engines for expert systems. This model cannot be used yet to provide the final diagnosis in developing hypertension in patients due that it requires clinician’s involvement for validation with real patients. However, this model can be used to make them aware that there is a probability that the patients could be developing hypertension.

Knowledge of the current model, and their parameters on risk-prediction models in general, is constructive to determine how to best approach the build of prediction models for hypertension, design the study, and interpret its results. When there is a realistic chance to find an applicable positive effect on decision-making and patient outcome, this model on a new setting could be potentially useful. This paper outlines the process for the development of a neural network risk prediction model, from choosing a data source and selecting features to assess model performance, performing validation, and assessing the impact of the model outcomes.

For future work, this model will be apply and re-train if necessary to a real balanced data set, and bigger network architectures will be considered. Also, new risk factors can be used to better handle the distribution and behavior of the input features for the model, and a sensitivity analysis will be performed to determine which inputs in our ANN model are significant with respect to the output. This paper will be the ground for the construction of a decision supporting tool that may be useful to healthcare practitioners for contributing to decision making about the risk of developing hypertension in typical or atypical patient screening circumstances.
